# Improving working memory abilities in individuals with Down syndrome: a treatment case study

**DOI:** 10.3389/fpsyg.2015.01331

**Published:** 2015-09-10

**Authors:** Hiwet Mariam Costa, Harry R. M. Purser, Maria Chiara Passolunghi

**Affiliations:** ^1^Department of Life Sciences, University of TriesteTrieste, Italy; ^2^School of Psychology, University of NottinghamNottingham, UK

**Keywords:** working memory, short term memory, Down syndrome, training, cognitive intervention

## Abstract

Working memory (WM) skills of individuals with Down’s syndrome (DS) tend to be very poor compared to typically developing children of similar mental age. In particular, research has found that in individuals with DS visuo-spatial WM is better preserved than verbal WM. This study investigated whether it is possible to train short-term memory (STM) and WM abilities in individuals with DS. The cases of two teenage children are reported: EH, 17 years and 3 months, and AS, 15 years and 11 months. A school-based treatment targeting visuo-spatial WM was given to EH and AS for six weeks. Both prior to and after the treatment, they completed a set of assessments to measure WM abilities and their performance was compared with younger typically developing non-verbal mental age controls. The results showed that the trained participants improved their performance in some of the trained and non-trained WM tasks proposed, especially with regard to the tasks assessing visuo-spatial WM abilities. These findings are discussed on the basis of their theoretical, educational, and clinical implications.

## Introduction

Down syndrome (DS) is a pervasive developmental disorder caused by abnormalities of chromosome 21. It is one of the most common causes of intellectual disability (ID), affecting about 1 in 700/1000 live births (Steele, 1996; Sherman et al., 2007; Parker et al., 2010). IQ generally ranges between 25 and 70 and the cognitive development of individuals with DS is characterized by significant delays and difficulties in working memory (WM) and short-term memory (STM) abilities. WM plays a key role in everyday life (e.g., reading, writing, arithmetic, learning, language-processing, orientation, and imagination) for typically developing (TD) children as much as for individuals with cognitive disabilities. Given this link between WM performance and classroom and daily life functioning, it is of substantial interest to investigate the effectiveness of interventions designed to reduce WM and STM difficulties in order to provide effective evidence-based training programs for young people with DS. Indeed, the enhancement of memory skills would be expected to promote skill development (e.g., Gathercole and Alloway, 2006) and independence of individuals with DS, minimizing the impact of the WM deficit on their lives.

### DS and WM Abilities

Working memory has been defined as a mental system that temporarily stores information while allowing that information to processed or manipulated (e.g., Baddeley, 1986). There are many different models of the structure of WM, but the investigation of WM abilities in DS has been largely conducted within the framework of the multi-component model of WM initially proposed by Baddeley and Hitch (1974; see also Baddeley, 1986, 2000). This model is composed of three main components. Two of these are the phonological loop and visual-spatial sketchpad, which are modality-specific systems dedicated to the passive storage of verbal and visuo-spatial information, respectively. The central executive, which in contrast is domain-general, controls the transfer of information to and from the two slave systems and it has been associated with a broad range of processing functions, such as inhibiting irrelevant information, shifting attention, and updating information. WM is considered an active system, involving both storage and processing, whereas STM involves only storage and no processing, as required in forward span tasks (Cornoldi and Vecchi, 2003; Swanson and Beebe-Frankenberger, 2004).

Working memory in DS has been investigated using a range of experimental approaches, providing substantial evidence of a dissociation between verbal and visuo-spatial abilities (Jarrold and Baddeley, 1997; Laws, 2002; Brock and Jarrold, 2005). Compared with children with ID or younger TD children matched for mental age, it has been found that there is a large deficit for those with DS in several verbal STM measures (Kay-Raining Bird and Chapman, 1994; Buckley et al., 1995; Laws, 1998; Jarrold et al., 1999). The current best explanation for the deficit in the phonological loop component of WM in individuals with DS is that they have a problem in storage itself, rather than in the encoding or rehearsal of information (Jarrold et al., 2002; Purser and Jarrold, 2005; Baddeley and Jarrold, 2007) Within Baddeley’s (1986) WM framework, DS seems to be associated with a reduction in phonological store capacity (Baddeley and Jarrold, 2007).

On the other hand, the visuo-spatial sketchpad abilities of individuals with DS are found to be in line with what one would expect given individuals’ general level of ability (Jarrold and Baddeley, 1997; Baddeley and Jarrold, 2007; Lanfranchi et al., 2012). Compared to TD children of the same mental age, DS children obtain largely equivalent scores (Lanfranchi et al., 2004). However, some studies showed that even if visuo-spatial STM was less impaired in DS than verbal STM, some differences emerged when the visuo-spatial component of WM was broken down into separate spatial and visual components (Ellis et al., 1989; Laws, 2002). Indeed, individuals with DS appear to show an unimpaired spatial memory (e.g., memory of spatial positions), but an impaired visual memory (memory of objects and their visual properties, such as colors, surfaces, etc.). Although visuo-spatial STM abilities seem to be better preserved if compared with phonological STM abilities, it is important to remember that both verbal and visuo-spatial WM skills are usually impaired if compared to chronological age-matched individuals (Kay-Raining Bird and Chapman, 1994).

The studies that examined the central executive component of WM suggested that there is a central executive limitation in DS. Children with DS have difficulties with executive load WM on both verbal and visuo-spatial measures, compared to mental age matched TD children (e.g., Lanfranchi et al., 2004). In particular, the results of a recent study of Lanfranchi et al. (2012) suggest that individuals with DS have a general executive deficit resulting in disproportionate deficits when two tasks are coordinated. These results are consistent with those of previous studies that also demonstrated such executive deficits in individuals with DS (Rowe et al., 2006; Lanfranchi et al., 2010) in addition to general difficulties in performing a variety of dual tasks (Lanfranchi et al., 2004).

### WM and Learning

A variety of studies have found that both verbal and visuo-spatial WM are strongly associated with a range of measures of learning (Gathercole and Baddeley, 1993; Jarvis and Gathercole, 2003; Gathercole and Alloway, 2006). Moreover, WM deficits are characteristic of children with learning difficulties both in literacy and in mathematics (Passolunghi and Siegel, 2001; Geary et al., 2004; Pickering, 2006; Schuchardt et al., 2008). Compared to WM abilities, STM skills are much more weakly associated with general academic attainment (Gathercole and Alloway, 2006). However, verbal STM skills are linked to reading progress and an accurate phonological representation within STM is required for new word learning (Service and Kohonen, 1995; Gathercole et al., 1997; Jarrold et al., 2009).

In the field of ID, some studies have suggested that the learning difficulties associated with DS might be underlain by difficulties in WM and STM. DS is characterized by generalized difficulties in performing number and calculation tasks (Marotta et al., 2006). In particular, individuals with DS exhibit several mathematical difficulties compared to TD individuals (Brigstocke et al., 2008). They obtain lower scores in a wide range of tests assessing basic mathematical knowledge, arithmetic abilities, and counting skills (Buckley and Sacks, 1986; Carr, 1988; Porter, 1999). Recently it has been suggested that visual WM memory difficulties in DS could lead to deficits in some early numerical abilities that are thought to be foundational to mathematical learning (Sella et al., 2013).

On the other hand, weak verbal WM and STM abilities make processing verbal information and learning from listening difficult for children with DS. Indeed, the marked phonological STM deficit seems to underlie the characteristic profile of language difficulties seen in individuals with DS (e.g., deficits in phonology, speech intelligibility, language production, syntax, reading; Dodd and Thompson, 2001; Byrne et al., 2002; Lanfranchi et al., 2009).

### WM Intervention

The results described above provide evidence that DS is characterized by significant delays and difficulties in WM and STM abilities that are associated with general learning disabilities and language impairment. Therefore, it is clearly of some importance to investigate the effectiveness of interventions designed to reduce the WM and STM difficulties, in order to provide effective evidence-based training programs for children with DS. However, WM has traditionally been considered a genetically fixed cognitive ability (Kremen et al., 2007). Therefore, it was not considered possible to enhance WM skills by acting on an individual’s environmental experiences and opportunities. Recently, a growing set of studies with TD children and adults have shown that WM skills can be improved through training demonstrating that considerable cerebral plasticity exists and that WM capacity may potentially be improved (Olesen et al., 2004; Thorell et al., 2009). Some studies have even shown a transfer effect of WM training on school-related skills (Holmes et al., 2009; St Clair-Thompson et al., 2010; Alloway et al., 2013; Passolunghi and Costa, 2014). However, the debate is still open and some authors questioned the effectiveness of WM training, arguing that there is currently too little evidence to conclude that such training generalizes to other cognitive skills (Melby-Lervåg and Hulme, 2013; Redick et al., 2015). Moreover, it has been emphasized that many studies that have examined the effect of WM training have not always applied adequate methodological criteria (e.g., no-contact control groups, single measures of cognitive constructs, inconsistent use of valid WM tasks, subjective measurement of change; Shipstead et al., 2010, 2012).

Given that the WM system is important for language learning, intervention studies designed to target the memory difficulties associated with DS have typically focused on improving verbal STM skills, generally by training children to use rehearsal strategies (Broadley and MacDonald, 1993; Laws et al., 1996). These studies have focused on improving the ability to repeat items in the correct order. Training in an overt cumulative rehearsal strategy has been shown to improve recall in groups with DS: such training involves the rehearsal, spoken aloud, of increasing amounts of information over the course of an STM task (Broadley and MacDonald, 1993; Comblain, 1994; Laws et al., 1996). Some of the studies dealing with rehearsal training used picture supports (children used visual processing to aid their memory span), with mixed findings for auditory span measures but clear improvements for measures of visual span (Broadley and MacDonald, 1993; Laws et al., 1996). A further study (Comblain, 1994) found a clear improvement in auditory memory span, beginning with picture supports, but phasing them out over the course of the task, ending in auditory-only training. Using a somewhat different approach Conners et al. (2008) used purely auditory rehearsal training and the results showed verbal span improvements.

To our knowledge, Bennett et al. (2013) is the only study to have investigated the effects of visuo-spatial training in DS children. This training consisted of seven computerized STM and WM games: four of them involved only the storage of visual information, two of them involved both manipulating and storing visual information, and one incorporated the storage of information in both modalities. Results showed that performance on trained and non-trained visuo-spatial STM tasks was significantly enhanced for children in the intervention group and this improvement was sustained four months later. However, they failed to find any transfer effect of the training either to visuo-spatial WM or verbal STM and WM skills. Despite this lack of transfer, these results suggest that training the visuo-spatial component of WM in a school setting may be possible for children with DS.

### The Present Study

The aim of the current study was to evaluate the efficacy of a school-based visuo-spatial WM training on STM and WM skills for two individuals with DS. Previous studies of memory training for individuals with DS have focused on the enhancement of verbal STM abilities by teaching rehearsal strategies, with positive results (Broadley and MacDonald, 1993; Comblain, 1994; Laws et al., 1996). Only one study has used WM training that taps both STM and WM skills (Bennett et al., 2013), in which a positive effect was found of training on visuo-spatial STM abilities (passive recall of information) but not on visuo-spatial WM abilities. However, several studies have demonstrated the effectiveness of WM training in both TD children and children with intellectual disabilities (Thorell et al., 2009; St Clair-Thompson et al., 2010; Van der Molen et al., 2010). Therefore, it was expected that the training, targeting visuo-spatial STM abilities (simple recall of information) and visuo-spatial WM abilities (ability to both simultaneously process and store information) would improve visuo-spatial WM and STM abilities. Moreover, it was expected that our training should improve not only the visuo-spatial component of WM, but also produce a transfer effect on the verbal component of WM. This hypothesis is in line with previous studies dealing with WM training in TD children and individuals with ID (Thorell et al., 2009; Van der Molen et al., 2010).

## Materials and Methods

### Participants

#### AS Case Report

AS is a boy with DS aged 15;11 at the time of the investigation. AS was selected from a database of participants, following on-going consent after recruitment for previous research studies by one of the authors (Harry R. M. Purser). After consent was provided by the schools, and prior to testing, parental consent was obtained. AS lives with his parents and attends a special secondary school for children with severe or moderate learning disabilities. AS was not on any medication at the time of the investigation. He received a diagnosis of DS 2 h after birth (confirmed trisomy 21, without mosaicism). He was born by cesarean section and his birth weight was 1.81 kg. AS has salivary gland malfunction and was hospitalized at 3 years old in order to receive surgical operation for the correction of umbilical hernia. Developmentally, sitting was normal at 0;7, though walking was late at 2;5. AS spoke his first words at 0;8 and did not start putting 2–3 words together until around 4–5 years. He received a diagnosis of dyspraxia at 5 years old and currently has some speech problems: he speaks in short, simplified sentences. AS attended a mainstream school from 2;6 to 12;0 when he moved to a school for children with learning disabilities. Before entering primary school, he never received any type of special education service or preschool support. AS was reported to enjoy school. He has problems with writing, but his general academic achievement is in line with what would be expected given his intellectual level. He was reported to be well behaved at school, and to have good relationships with both adults and peers. AS was also reported to enjoy sports, in particular swimming. Additionally, he enjoyed 2 years work experience in a garden center.

Non-verbal Intelligence was assessed at time of testing using Raven’s Colored Progressive Matrices (RCPM; Raven et al., 1998). AS’s RCPM raw score was 16, and his non-verbal mental age was 7. AS was also assessed on the British Picture Vocabulary Scale III (BPVS; Dunn et al., 2009), a measure of receptive vocabulary. AS BPVS raw score was 96, his vocabulary mental age was 6 years and 5 months.

#### EH Case Report

EH is a girl with DS aged 17;3 at the time of the investigation. Selection and consent were via the procedures described for AS. EH lives with her parents and attends a special secondary school for children with severe or moderate learning disabilities. EH was not on any medication at the time of the investigation except for hay fever tablets. She received a diagnosis of DS immediately after birth (confirmed trisomy 21, without mosaicism). She was born naturally and birth weight was 2.72 kg.

Developmental milestones were reportedly delayed: she started sitting at 0;10 and walking at 2;5. EH spoke her first words at 0;7 and did not start putting 2–3 words together until she was 3;0. Currently EH was not reported to have any speech problem. EH attended a mainstream school until 11;0 when she moved to a school for children with learning disabilities. Prior to entering primary school she never received any type of special education service or preschool support. She was reported to enjoy school with normal reading, spelling and arithmetic skills. EH was also reported to be well behaved at school, even if sometimes she does not want to do her homework. She gets on well both with both adults and peers. EH was reported to enjoy music and dance.

Non-verbal Intelligence was assessed at time of testing using RCPM (Raven et al., 1998). EH’s RCPM raw score was 19, and her non-verbal mental age was 8. EH was also assessed on the BPVS III (Dunn et al., 2009), a measure of receptive vocabulary. AS BPVS raw score was 101, her vocabulary mental age was 7 years.

#### TD Control Group

The TD group was comprised of children randomly selected on the basis of date of birth from a mainstream primary school. Both school and parental consent were obtained prior to testing. The WM training used in this study targeted visuo-spatial WM, and AS and EH were therefore matched to TD controls on the basis of non-verbal intelligence assessed with the RCPM test. Given that the RCPM test is commonly used to estimate of IQ (Belacchi et al., 2010; Lanfranchi and Carretti, 2012; Kolkman et al., 2013; Xenidou-Dervou et al., 2014), this matching criteria ensured that performance differences prior and after the training were not due to any general intelligence differences. Children with a RCPM score below 15 and greater than 21 were excluded to ensure that AS and EH were compared to children with a comparable non-verbal intelligence. Children with statements of special educational need (as identified by local educational services) were excluded. There were 17 TD children (eight boys and nine girls) in the TD group. The mean age was 6 years, 1 (SD 0 years, 7 months), with a range of 5 years 7 months to 7 years 0 month.

### Procedure

Participants were individually tested at school in two sessions separated by approximately 1 week. Testing sessions lasted approximately 30 min. For matching purposes, the participants with DS completed their testing session first. Then, based on the score reached at the RCPM test, the 17 TD children were selected and they completed their testing sessions.

The WM training undertaken by the participants with DS included eight of paper-and-pencil tasks that were designed to improve visuo-spatial WM abilities. Over six successive weeks, AS and EH participated in 12 training sessions (twice weekly). In each session, two games were played. Training duration was 40 min per session. After the training, AS and EH’s WM abilities were assessed again. In all the assessment tasks the child was given an example of how to perform the trial before to start. Only when the child understood the instructions the task was recorded.

### Assessments

#### Visuo-Spatial STM

Pathway recall (Lanfranchi et al., 2004). The child was shown a path taken by a small toy frog on a 3 × 3 or 4 × 4 grind. The child had to recall the pathway immediately after presentation by moving the frog from square to square, reproducing the experimenter’s moves. The task is composed of eight trials and had four levels of difficulty, depending on the number of steps in the frog’s path and dimensions of the chessboard (3 × 3 in the first level with two steps and 4 × 4 in the other levels, with two, three, and four steps, respectively). Two trials for each difficulty level were presented. A score of 1 was given for every trial performed correctly. The minimum score was 0 and the maximum was 8.

#### Visuo-Spatial WM

Pathway recall backward (Lanfranchi et al., 2004). The child was shown a path taken by a small toy frog on a 3 × 3 or 4 × 4 grind, in the same way as the pathway recall task. The child had to remember the path in the reverse order. There were four levels of difficulty, depending on the number of steps in the frog’s path and the size of the chessboard (3 × 3 in the first and second levels, and 4 × 4 in the other levels). Two trials for each difficulty level were presented. A score of 1 was given for every trial performed correctly. The minimum score was 0 and the maximum was 8. Selective pathways task (Lanfranchi et al., 2004). The child was shown one or two small toy frog’s paths taken by the frog on a 4 × 4 grind, as in earlier tasks. The child had to remember the frog’s starting position(s). The task had four different levels of difficulty, depending on the number of pathways and the number of steps in each pathway. There were two trials for each difficulty level. At levels one and two, respectively, one pathway with two steps and one with three steps was presented. At levels three and four, two pathways of two and three steps, respectively, were presented. A score of 1 was given for every trial performed correctly. The minimum score was 0 and the maximum was 8. Visuo-spatial dual task (Lanfranchi et al., 2004). The child had to remember the frog’s starting position on a path on a 4 × 4 grind, in which one of the 16 cells was red. The child also had to tap on the table when the frog jumped onto the red square. The task had four different levels of difficulty, depending on the number of steps in the path (i.e., two, three, four, and five steps, respectively). Two trials for each difficulty level were presented. The score of 1 was given for every trial performed correctly, with the child both remembering the first position of the pathway and performing the tapping task. Otherwise, a score of 0 was given. The minimum score was 0 and the maximum score was 8.

#### Verbal STM

Forward word recall (Lanfranchi et al., 2004). In this task lists of two to five words were presented to the child, who was required to repeat the list immediately and in the same order of presentation. Two trials for each difficulty level were presented. A score of 1 was given for every trial performed correctly. The minimum score was 0 and the maximum was 8.

#### Verbal WM

Backward word recall (Lanfranchi et al., 2004). Lists of two to five words were presented, and the child was asked to repeat each list in reverse order immediately after presentation. Two trials for each difficulty level were presented. A score of 1 was given for every trial performed correctly. The minimum score was 0 and the maximum was 8.

Selective word recall (Lanfranchi et al., 2004). One or two lists were presented to the child, who was required to repeat the first word of each list after the presentation of the entire series. There were four difficulty levels, depending on the number of lists (one or two) and the number of words (two or three) in each list. Two trials for each difficulty level were presented. At levels one and two, respectively, one list with two words and one with three words were presented. At levels three and four, two lists with two and three words, respectively, were presented. A score of 1 was given for every trial performed correctly. The minimum score was 0 and the maximum was 8.

Verbal dual task (Lanfranchi et al., 2004). The child was presented with a list of two to five two-syllable words and was asked to recall the first word on the list and tap on the table when the target word (“ball”) was presented. The test is made up by eight trials, two for each of the four difficulty levels. A score of 1 was given for every trial performed correctly, when the initial word of the sequence was remembered correctly at the same time the dual task was performed. Otherwise, a score of 0 was given. The minimum score was 0 and the maximum was 8.

### Visuo-Spatial WM Training

The visuo-spatial WM training used was an adapted version of a WM training used in a previous study (Passolunghi and Costa, 2014) and it included different tasks that were designed to enhance visuo-spatial STM and WM abilities. The training was implemented for six weeks, twice weekly, with each session lasting 40 min. The full training program consisted of eight different games grouped into two different categories: four visuo-spatial WM games, and four visuo-spatial STM games. In each session, two games were played: one mainly focused on the enhancement of visuo-spatial STM, one mainly focused on the enhancement of visuo-spatial WM.

The training was adaptive with the instructor adapting the tasks to the child’s performance (e.g., if the child failed to remember three items, on the next occasion the instructor asked for two items and, after a successful repetition of two items, asked for three again). This procedure allows to individualize the intervention by constantly assessing children’s performance and adapting the difficulty level of the task, thus maintaining each child in his or her zone of proximal development (Vygotsky, 1978). The instructor gave continuous feedback to the children during the training. The children participated in the activity one after the other.

#### Visuo-Spatial STM Games

The first category tapped visuo-spatial STM abilities. These games required the immediate serial recall of visuo-spatial information. For the game “Farmers,” a 1.5 m × 1.5 m matrix with 25 elements positioned on the floor was used. The instructor presented paths of different lengths on the matrix. Steps were presented at the rate of approximately one step every 2 s. Children had to repeat the steps of the path in the presented order. In the game “Circles” 25 hula hoops were randomly positioned on the floor. The instructor presented paths of different lengths on the circles. Steps were presented at the rate of approximately one step every 2 s. Children had to repeat the steps in the presented order. In the “Game of cards,” 7 × 10 cm cards with pictures (animals, fruit & vegetables, and objects) were presented, one at a time at the rate of a card per second, and the children had to recall the list in the correct order using cards with pictures to respond. In the “Game of numbers” 7 × 10 cm cards with numbers were presented, one at a time at the rate of a card per second, and the children had to recall the list in the correct order using cards with numbers to respond.

#### Visuo-Spatial WM Games

The second category of games tapped visuo-spatial WM abilities. These games required a dual task procedure (“Colors” and “Pairs”) or a backward recall (“The farmers backward” and “Game of Cards Back”).

For the game “Colors” A 1.5 m × 1.5 m matrix with 25 colored elements (blue, yellow, red, green, and black) was positioned on the floor. The instructor presented paths of different lengths on the matrix. Children had to name the color of each element during the presentation of the path and then recall the first step of the path after presentation. The game “Pairs” challenged the children to remember the locations of 7 × 10 cm cards with pictures (animals, fruit & vegetables, and objects) placed on a grid. On each turn, a player turns over two cards (one at a time) and keeps them if they match. For the game “Farmers backward,” a 1.5 m × 1.5 m matrix with 25 elements positioned on the floor was used. The instructor presented paths of different lengths on the matrix. Steps were presented at the rate of approximately one step every 2 s. Children had to repeat the steps of the path in the reverse order after presentation. In the “Game of Cards Back,” some 7 × 10 cm cards with pictures (animals, fruit & vegetables, and objects) were presented, one at a time at the rate of a card per second, and the children had to recall the list in the reverse order using pictures to respond.

### Analysis

Crawford and Howell’s (1998) modified *t*-test was used to test whether the difference between the single cases (AS and EH) and the control sample was statistically different. This method provides both significance tests and a point estimate of the percentage of the population that would obtain a more extreme score (or different score) and an interval estimate (i.e., confidence limits) on this percentage. The effect size (z_cc_) and 95% confidence interval around the effect size were also calculated using the methods proposed by Crawford et al. (2010). Analyses were run using the program Singlims_ES.exe, an upgraded version of the program Singlims.exe (Crawford and Garthwaite, 2002). It implements classical methods for comparison of a single case’s score to scores obtained in a control sample.

In agreement with Perneger (1998), Bonferroni adjustments were not applied. If using Bonferroni adjustments for small sample sizes, the interpretation of a finding becomes dependent upon the number of analysis performed so they automatically increase the likelihood of Type II errors and important performance differences may be missed (Perneger, 1998).

The focus of the current study was of individuals with DS. It was therefore expected that where performance differed to that of controls would be in the direction of impaired performance and one tailed *t*-test were used for the analysis (Crawford et al., 2003). However, literature shows how the WM memory deficit seems to be limited to the verbal rather than visuo-spatial domain (Jarrold and Baddeley, 1997; Laws, 2002). Indeed, the visuo-spatial sketchpad abilities of individuals with DS seems to be in line with what one would expect given individuals’ general level of ability. (Jarrold and Baddeley, 1997; Baddeley and Jarrold, 2007; Lanfranchi et al., 2012). Therefore, for visuo-spatial STM measures two-tailed *t*-tests were used. For all *t*-tests, the 0.05 probability level for significance was used.

## Results

**FIGURE 1:**
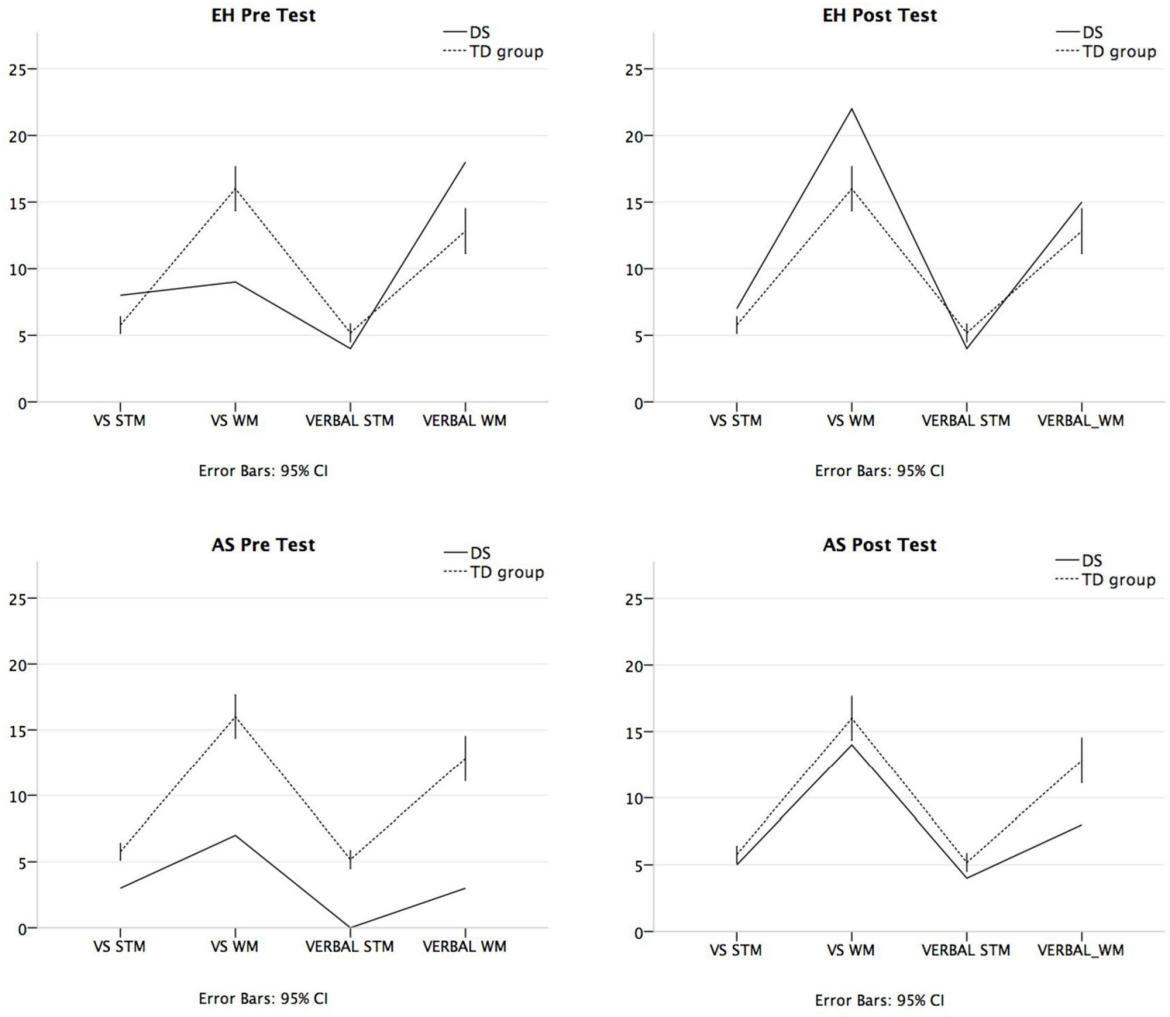
**Visuo-spatial STM and cumulative working memory (WM) scores, and verbal short-term memory (STM) and cumulative WM scores at pre-testing (for EH, AS, and TD group) and post-intervention (for EH and AS).** VS STM, Visuo-spatial STM score; VS WM, Cumulative visuo-spatial WM score; VERBAL STM, Verbal STM score; VERBAL WM, Cumulative verbal WM score.

Performance prior and after training is reported for EH and AS, two teenagers with DS, in comparison to matched TD controls (**Table 1**). In the first part of this section, results in visuo-spatial STM and WM abilities are reported. In the second part of the section, the results in verbal STM and WM are reported. Both parts are followed by a summary of the main findings (see also **Figure 1**).

**Table 1:** Scores for the working memory (WM) and short-term memory (STM) measures.

		TD group (*n* = 17)		AS				EH			
	Task	Mean	(*SD*)	Pre	*Z_CC_* (95% CI)	Post	*Z_CC_* (95% CI)	Pre	*Z_CC_* (95% CI)	Post	*Z_CC_* (95% CI)
Verbal measures	Word span (STM)	5.18	1.38	0	–3.75 (–5.12 to –2.37)	4^∗^	–0.85 (–1.40 to –0.29)	4	–0.85 (–1.40 to –0.29)	4	–0.85 (–1.40 to –0.29)
	Word span backward	3.00	0.87	0	–3.45 (–4.71 to –2.17)	0	–3.45 (–4.71 to –2.17)	3	0.00 (–0.47 to 0.47)	4	1.14 (0.52 to 1.76)
	Selective word recall	5.12	1.36	3	–1.56 (–2.26 to –0.83)	6	0.64 (0.11 to 1.16)	8	2.12 (1.24 to 2.98)	6	0.65 (0.11 to 1.16)
	Verbal dual task	4.71	2.42	0	–1.95 (–2.76 to –1.12)	2^∗^	1.12 (–1.72 to –0.50)	7	0.95 (0.36 to 1.51)	5	0.12 (–0.36 to 0.59)
	Cumulative verbal WM	12.82	3.34	3	–2.94 (–4.05 to –1.82)	8^∗^	–1.44 (–2.12 to –0.75)	18	1.55 (0.83 to 2.25)	15	0.65 (0.12 to 1.17)
Visuo-spatial measures	Pathway recall (STM)	5.76	1.30	3	–2.12 (–2.98 to –1.24)	5^∗^	–0.58 (–1.09 to –0.06)	8	1.72 (0.95 to 2.47)	7	0.95 (0.37 to 1.52)
	Pathway recall backward	5.47	1.23	4	–1.19 (–1.81 to –0.56)	4	–1.19 (–1.81 to –0.56)	5	–0.38 (–0.87 to 0.12)	7	1.24 (0.59 to 1.87)
	Selective pathways	5.06	1.75	3	–1.18 (–1.79 to –0.54)	6	0.54 (0.02 to 1.04)	1	–2.32 (–3.24 to –1.38)	8^∗^	1.68 (0.92 to 2.42)
	Visuo-spatial dual task	5.47	1.70	0	–3.22 (–4.41 to –2.01)	4^∗^	–0.86 (–1.42 to –0.29)	3	–1.45 (–2.13 to –0.75)	7	0.90 (0.32 to 1.46)
	Cumulative visuo-spatial WM	16.00	3.28	7	–2.74 (–3.79 to –1.68)	14^∗^	–0.61 (–1.12 to –0.08)	9	–2.13 (–3.00 to –1.25)	22^∗^	1.83 (1.03 to 2.61)

*^∗^Improvement from a significantly impaired performance in the pre-test to a score that didn’t differ significantly from the performance of TD group. *Z_CC_* = effect size index for the difference between the case and controls.*

### Visuo-Spatial STM

#### Pathway Recall

EH’s *Pathway recall* score did not differ significantly from the TD group either in the pre-test, *t* = 1.67, *p* = 0.11, or in the post-test, *t* = 0.93, *p* = 0.37. In both sessions her score was higher compared to the mean score of the control TD group and in the pre-test her performance was at ceiling. The estimated percentage of normal population falling below case’s score was 94.33% (95% CI: 82.98%; 99.33%) before training and was 81.61% (95% CI: 64.29%; 93.59%) after the training.

Prior to training, AS recalled significantly fewer paths than the control group, *t* = 2.18, *p* = 0.04. After training AS improved in performance on the *Pathway recall* and in the post-test session there was no longer a significant difference from the TD group, *t* = 0.60, *p* = 0.56. The estimated percentage of the normal population falling below the case’s score was 2.78% (95% CI: 0.01%; 10.69%) before training and increased up to 28.89% (95% CI: 13.72%; 47.57%) after the training.

### Visuo-Spatial WM

#### Pathway Recall Backward

EH’s score in *Pathway recall backward* did not differ significantly from the TD group in the pre-test session, *t* = 0.37, *p* = 0.36, or the post-test session, *t* = 1.2, *p* = 0.12. However, the estimated percentage of the normal population falling below the case’s score increased from 35.76% (95% CI: 19.22%; 54.64%) before training up to 87.79% (95% CI: 72.37%; 96.94%) after the training.

For AS, the score was the same prior and after the training and his performance did not differ significantly from the TD group, *t* = 1.16, *p* = 0.13. The estimated percentage of the normal population falling below the case’s score was 13.12% (95% CI: 3.49%; 28.90%).

#### Selective Pathways

EH’s *Selective pathways* performance in pre-test session was significantly impaired compared to the TD group, *t* = 2.25, *p* = 0.02. Her performance improved after the training with a post-test score at ceiling and higher than the mean score of the TD group, *t* = 1.63, *p* = 0.06. Strikingly, the estimated percentage of the normal population falling below the case’s score was 1.92% in the pre-test (95% CI: 0.06%; 8.3%) and 93.90% (95% CI: 82.17%; 99.22%) in the post-test.

AS’s performance did not differ significantly from the TD group in the pre-test session, *t* = 1.14, *p* = 0.13, or the post-test session, *t* = 0.52, *p* = 0.30. The estimated percentage of the normal population falling below the case’s score was 13.47% (95% CI: 3.67%; 29.38%) before training and was 65.56% (95% CI: 50.80%; 85.08%) after the training.

#### Visuo-Spatial Dual Task

In the *Visuo-spatial dual task*, EH’s performance did not differ significantly from the TD group in the pre-test session, *t* = 1.41, *p* = 0.09, or the post-test session, *t* = 0.87, *p* = 0.20. However, the estimated percentage of the normal population falling below the case’s score increased from 8.85% in the pretest (95% CI: 1.65%; 22.57%) to 80.26% (95% CI: 62.67%; 92.75%) in the post-test.

AS’s *Visuo-spatial dual task* performance in the pre-test session was significantly impaired compared to the TD group, *t* = 3.13, *p* = 0.003, since he was not able to perform the double task. After training, AS’s score did not differ significantly from the TD group, *t* = 0.84, *p* = 0.21. The estimated percentage of the normal population falling below the case’s score was 0.32% (95% CI: 0.00%; 2.23%) before training and increased up to 20.65% (95% CI: 7.84%; 38.42%) after the training.

#### Cumulative Visuo-Spatial WM Score

In order to better understand the nature of EH and AS’s WM improvements and for data reduction purposes, a *Cumulative visuo-spatial WM score* was created by summing the scores of the *Visuo-spatial dual task*, the *Selective pathways*, and *Pathway recall backward.*

EH’s *Visuo-spatial WM cumulative score* prior to training was significantly impaired compared to the TD group, *t* = 2.07, *p* = 0.03. After training, EH’s performance increased (EH = 22, control mean = 16, SD, 3.28) and she obtained a significantly higher score than the TD group, *t* = 1.77, *p* = 0.047. The estimated percentage of the normal population falling below the case’s score was 2.73% (95% CI: 0.014%; 10.55%) before training. After the training, the results showed that the estimated percentage of the normal population falling below EH’s score was 95.28% (95% CI: 84.87%; 99.54%).

AS’s *Visuo-spatial WM cumulative* performance in the pre-test session was significantly impaired compared to the TD group, *t* = 2.67, *p* = 0.008. After the training, there was no longer a significant difference from the TD group, *t* = 0.59, *p* = 0.28. The estimated percentage of the normal population falling below the case’s score was 0.84% (95% CI: 0.007%; 4.64%) before training and was 28.09% (95% CI: 13.11%; 46.71%) after the training.

### Summary

EH’s performance in visuo-spatial STM, assessed with the pathway recall task was higher compared to the mean score of the control TD group both in the pre-test and post-test. The results did not show an improvement of EH’s visuo-spatial STM abilities after training. Her lower performance in the post-test session was probably be due to a regression to the mean effect.

Considering the tasks assessing visuo-spatial WM abilities, in the *Pathway recall backward* and in the *Visuo-spatial dual task* EH performance did not differ significantly from the TD group either in the pre-test or post-test sessions. However, in both tasks there was an improvement of performance after the training, as shown by the increased estimated percentage of the normal population falling below the case’s score in the post-test session (from 35.76 to 87.78% for the *Pathway recall backward*; from 16.80 to 72.62% in the *Visuo-spatial dual task).* The third task used in order to assess visuo-spatial WM abilities was the *Selective pathways.* EH’s performance in the pre-test session was significantly impaired compared to the control TD group. The results showed that her performance improved after the training and her score did not differ from the TD group.

If one considers the *Visuo-spatial WM cumulative score*, EH’s performance prior to training was significantly impaired compared to the control group. The training led to an improvement of overall visuo-spatial WM abilities given that after the training EH obtained a significant higher score in comparison to the TD group.

AS’s performance in visuo-spatial STM, assessed with the pathway recall, was significantly impaired compared to the control TD group in the pre-test session. The results showed that his performance improved after the training when the score did not differ from the TD group.

Considering the tasks assessing visuo-spatial WM abilities, AS’s *Pathway recall backward* performance prior to training did not differ significantly from the TD group. Results showed no improvements in the post-test session. In the *Selective pathways* AS’s performance did not differ significantly from the TD group either in the pre-test or post-test session. However, there was an improvement of performance after the training as shown by the increased estimated percentage of the normal population falling below the case’s score in the post-test session (from 13.47 to 65.56%). Regarding the *Visuo-spatial dual task*, AS showed impaired performance in the pre-test session. The performance improved after the training, with no more significant difference from the average scores obtained by the TD group.

If one considers the *Visuo-spatial WM cumulative score*, AS’s performance prior to training was significantly impaired compared to the control group. The training led to an improvement of overall visuo-spatial WM abilities, given that after the training there was no longer a significant difference from the TD group.

### Verbal STM

#### Word Span

For EH, *word span* score was the same prior and after the training and her performance did not differ significantly from the TD group, *t* = 0.83, *p* = 0.21. The estimated percentage of normal population falling below case’s score was 20.91% (95% CI: 8.01%; 38.72%).

AS’s *word span* performance in pre-test session was significantly impaired compared to the TD group, *t* = 3.65, *p* = 0.001, since it was not able to perform the task. After training, AS’s score did not differ significantly from the TD group, *t* = 0.83, *p* = 0.21. The estimated percentage of the normal population falling below the case’s score was 0.11% (95% CI: 0%; 0.88%) before training and increased up to 20.21% (95% CI: 8.01%; 38.72%) after the training.

### Verbal WM

#### Word Span Backward

EH’s *Word span backward* score in the pre-test was equal to the average score obtained from the control TD group, *t* = 0, *p* = 0.50. In the post-test session again EH’s performance did not differ significantly from the TD group, *t* = 1.17, *p* = 0.14. The estimated percentage of the normal population falling below the case’s score was 50.00% (95% CI: 31.73%; 68.27%) before training and was 85.98% (95% CI: 69.87%; 96.05%) after the training.

AS was not able to perform the *Word span backward* either before or after the training. His performance was significantly poorer than the control group, *t* = 3.35, *p* = 0.002, and the estimated percentage of the normal population falling below AS’s score was 0.20% (95% CI: 0%; 1.51%).

#### Selective Word Recall Task

EH’s *Selective word recall* performance in the pre-test session was at ceiling and significantly higher than the TD group, *t* = 2.06, *p* = 0.03 while EH’s post-test performance did not differ significantly from the TD group, *t* = 0.63, *p* = 0.27. The estimated percentage of the normal population falling below the case’s score was 85.98% (95% CI: 69.87%; 96.05%) in the pre-test and was 73.08% (95% CI: 54.53%; 87.77%) in the post-test.

The difference between AS’s *Selective word recall* performance and the TD group did not differ significantly from the mean score of the TD group either in the pre-test, *t* = 1.51, *p* = 0.07, or post-test, *t* = 0.63, *p* = 0.26. However, it can be seen that the effect size for the case’s difference is quite large in the pre-test: the case’s difference is over 1.5 SD from the mean difference in controls. After training, AS’s *Selective word recall* score was higher compared to the mean score of the control TD group. The estimated percentage of the normal population falling below the case’s score was 7.46% (95% CI: 1.18%; 20.27.77%) before training and increased up to 73.08% (95% CI: 54.54%; 87.77%) after the training.

#### Verbal Dual Task

EH’s *Verbal dual task* score did not differ significantly from the mean score of the TD group either in the pre-test, *t* = 0.92, *p* = 0.18, or post-test, *t* = 0.12, *p* = 0.45. The estimated percentage of the normal population falling below EH’s score was 81.43% (95% CI: 64.07%; 93.47%) in the pre-test and was 54.55% (95% CI: 35.97 %; 72.41%) in the post-test.

AS’s *Verbal dual task* performance in the pre-test session was significantly impaired compared to the TD group, *t* = 1.89, *p* = 0.038, since he was not able to perform the double task. After training, AS’s score did not differ significantly from the TD group, *t* = 1.09, *p* = 0.15. The estimated percentage of the normal population falling below the case’s score was 3.84% (95% CI: 0.29%; 13.22%) before training and was 14.63% (95% CI: 4.26%; 30.93%) after the training.

#### Cumulative Verbal WM

To better understand the nature of EH and AS’s WM abilities and for data reduction purposes a *Cumulative verbal WM score* was created by summing the scores of the *Verbal dual task*, the *Selective word recall*, and *Word span backward.*

EH’s *Cumulative verbal* score in both sessions was higher compared to the mean score of the control TD group. Her score was higher compared to the TD group in the pre-test, but the difference was not significant, *t* = 1.50, *p* = 0.07. In the post-test, there was a decrease of performance but her score remained higher than the average score of the TD group. EH’s post-test performance did not differ significantly from the TD group, *t* = 0.63, *p* = 0.27. The estimated percentage of the normal population falling below the case’s score was 92.44% (95% CI: 79.56%; 98.79%) before training and was 73.26% (95% CI: 54.72%; 87.90%) after the training.

AS’s *Cumulative verbal WM* score in the pre-test session was significantly impaired compared to the TD group, *t* = 2.86, *p* = 0.006. After the training, there was no longer a significant difference from the TD group, *t* = 1.40, *p* = 0.09. The estimated percentage of the normal population falling below the case’s score was 0.56% (95% CI: 0.0073%; 3.46%) before training and was 8.99% (95% CI: 1.70%; 22.80%) after the training.

### Summary

EH’s performance in verbal STM, assessed with the *Word span*, did not differ significantly from the TD group prior to training. Results showed no improvements in the post-test session.

Considering the tasks assessing verbal WM abilities, the results showed no impairments in any verbal WM measure compared to the TD group in the pre-test session. After the training period the performance in all verbal WM tasks (*Word span backward, Selective word recall*, and *Verbal dual task*) remained within the range of the TD group. In *Selective word recall* and in the *Verbal dual task* there was a decrease of performance, but her score remained higher than the average score of the TD group both in the pre- and post-test sessions. Only in the *Word span backward task* was there an increased performance at post-test, as shown by the increased estimated percentage of the normal population falling below the case’s score in the post-test session (from 50.00% in the pre-test to 85.98% in the post-test).

If one considers the *Verbal WM cumulative score*, EH’s performance did not differ significantly from the TD group either in the pre-test or in the post-test. The results show a lower performance in the post-test but it should be noted that in both sessions her score was higher than the mean score of the control TD group.

EH lower performances in the the post-test session compared to the pre-test session in some of the tasks (*Selective word recall, Verbal dual task, and Verbak WM cumulative score*) could be due to a regression to the mean effect. Ideeed, she showed a high performance in the pre-test, and even if her performance decreased in the post-test, in both sessions was higher compared to the mean score of the control TD group.

AS’s performance in all verbal STM and WM tasks was significantly impaired compared to the control TD group in the pre-test session, except for *Selective word recall* where the performance difference relative to the TD group was not significant. The results showed that AS’s verbal STM performance improved after the training when his score did not differ from the TD group.

Considering the tasks assessing verbal WM abilities, the results showed an improvement in the post-test session in the *Selective word recall* and in the *Verbal dual task*, with no significant difference from the average scores obtained by the TD group. AS was not able to perform the *word span backward* either before or after the training.

If one considers the *Verbal WM cumulative score*, AS’s performance prior to training was significantly impaired compared to the control group. The training lead to an improvement of overall Verbal WM abilities, given that after the training there was no longer a significant difference from the TD group.

## Discussion

The aim of our study was to evaluate the impact of a school-based visuo-spatial WM training on the STM and WM skills of two individuals with DS. With regard to visuo-spatial abilities, both EH’s and AS’s visuo-spatial WM cumulative scores (created by summing the scores of the *Visuo-spatial dual task*, the *Selective pathways*, and *Pathway recall backward)* improved after the training. Indeed, while in the pre-test their performance was significantly impaired compared to the TD group, in the post-test session their scores did not differ significantly from the performance of TD group. EH’s scores were improved in all visuo-spatial WM tasks after training. In particular, her performance in the *Selective pathways* was significantly impaired in the pre-test, while after training there was no longer significant difference from the TD group. AS improved his performance in all the visuo-spatial WM tasks after training except for the *Pathway recall backward* task that, in any case, remained within the range of the TD group. In particular, his performance in the *Visuo-spatial dual task* was significantly impaired in the pre-test while after the training there was no longer a significant difference from the TD group. Moreover, AS’s Pathway recall performance (visuo-spatial STM) was significantly impaired in the pre-test while after the training there was no longer a significant difference from the TD group.

It should be noted that both EH and AS significantly improved their visuo-spatial scores after training, mostly on those tasks on which they were significantly impaired in the pre-test session. These results suggest that our training successfully enhanced visuo-spatial abilities, improving also those skills in which they were deficient in the pre-test compared to the TD group.

On the basis of the results of previous studies (Thorell et al., 2009; Van der Molen et al., 2010) and given that our visuo-spatial WM training included complex memory tasks involving the central executive component of WM, a transfer of improvements to the verbal domain was expected. The results showed that AS’s verbal STM and WM skills were significantly impaired compared to the control TD group prior to training. After the training his performance improved and there was no longer a significant difference from the TD group, except for the *Word span backward* score. EH’s performance did not differ significantly from the TD group in any verbal STM and WM task, either in the pre- or post-test session. There was no improvement from pre-test to post-test except for the *Word span backward*. Therefore, there was a transfer of the visuo-spatial WM training effects on verbal abilities for AS, while EH didn’t showed any significant improvement in her verbal STM or WM performance. This result could be explained considering the different profiles of the participants, which reflect the wide variation in the effects of the chromosomal abnormality on the development in the DS. In the pre-test assessment, AS showed a generally weak profile, with most of the verbal and visuo-spatial scores significantly below the mean of the TD group. In contrast, EH showed a stronger profile with all the verbal scores and most of visuo-spatial scores within the range of the TD group. Moreover, EH’s scores in all verbal WM measures (*Word span backward, Selective word recall, Verbal dual task, Verbal WM cumulative score*) both in the pre-test and in the post-test were equal or higher than the average scores of the TD control group. Taken together, these results indicate that the training had a beneficial effect, especially on those skills that were deficient (below expected standards), while it is more difficult to influence those skills that are already in line with what one would expect given individual’s general level of ability.

To explain the stronger memory profile of EH, it can be hypothesized that her good education path/career and her good verbal abilities encouraged the development of WM skills. In particular, participation in school activities may have led to a familiarity in processing verbal information. On the other hand, AS’s dyspraxia and speech problems could explain his general low WM and STM profile (Alloway and Archibald, 2008).

There are some limitations of the study. First, although we administered WM tasks used with individuals with Down syndrome (7–23 years) in previous studies (Lanfranchi et al., 2004, 2010, 2012) we found some ceiling performance levels with EH in the pre-test and in the post-test session. The ceilings for EH are probably connected to her stronger memory profile as explained above and may be prevented in future studies by using a more complex version of the same WM tasks. Second, only two single case treatments were studied. While the results are encouraging, extension with further data is required to better assess the effectiveness of the WM training outlined. A further limitation is that changes were only assessed immediately after the training so that there is no information about the longer-term stability of any training-related gains in performance. Previous studies reported an increased affects of WM training at follow-up compared with immediate effects in the post-test (Klingberg et al., 2005; Holmes et al., 2009; Van der Molen et al., 2010). It should be important in future studies to follow up post-intervention to see whether benefits of training last and to investigate the effectiveness of this kind of WM training with group studies.

The findings of the present study are promising and could have important practical implications for intervention. In fact, the training program successfully enhanced AS’s and EH’s WM, a central and important cognitive aspect for classroom and daily life functioning. Our results, in line with previous studies (Klingberg et al., 2002; Thorell et al., 2009; Van der Molen et al., 2010), provide further evidence that WM abilities can be improved and that school-based visuo-spatial memory training can be effective for children with DS, also without the support of a computer. Given the importance of WM abilities for the development of a broad range of learning achievement (e.g., Alloway and Alloway, 2010), further work is required to investigate possible transfer effects of visuo-spatial WM training on learning in individuals with DS.

## Conflict of Interest Statement

The authors declare that the research was conducted in the absence of any commercial or financial relationships that could be construed as a potential conflict of interest.
